# Multimodality management of leiomyosarcoma of the cervix

**DOI:** 10.3332/ecancer.2018.830

**Published:** 2018-04-30

**Authors:** Vijaya Kumar Jayaram, J Parikshith, Geeta Sowmya Narayanan, Richa Tiwari, R Veena, S Prathima, MS Ganesh, Chattakonda Sai Snehith, Esther Praisy

**Affiliations:** 1 Department of Radiation Oncology, Vydehi Institute of Medical Sciences and Research Centre, Bengaluru 560066, India; 2 Department of Pathology, HCG Oncology Centre, Bengaluru 560013, India; 3 Department of Pathology, Vydehi Institute of Medical Sciences and Research Centre, Bengaluru 560066, India; 4 Department of Surgical Oncology and Oncology, Vydehi Institute of Medical Sciences and Research Centre, Bengaluru 560066, India

**Keywords:** leiomyosarcoma, young age, multimodality management

## Abstract

We present the case of a young female patient who presented to the outpatient department with a history of bleeding per vagina, diagnosed with leiomyosarcoma of the cervix; the patient underwent total abdominal hysterectomy with pelvic lymph node dissection. In this article, we mainly discuss multimodality therapy in the management of an unusual variety of tumour in the uterine cervix.

## Introduction

Leiomyosarcoma is one of the rare histopathological variants of tumours of the cervix comprising nearly 1% of overall tumours of the cervix. Diagnosis is made on the basis of histopathological findings and immunohistochemical analysis. The treatment of choice in such patients is a triple therapy comprising surgery, chemotherapy and radiation therapy [1].

## Case study

**Figure 1(A–C).**
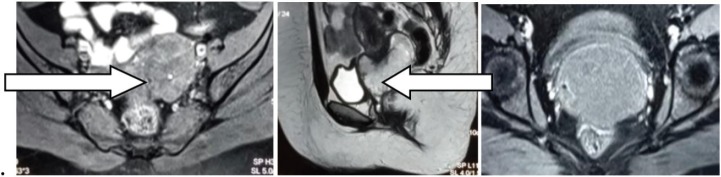
MRI of the pelvis; T2W and SPAIR hyper-intense polypoidal mass arising from the cervix measuring 6.2 × 6.9 × 7.2 cm^3^.

A twenty-year-old female patient presented with a history of increased blood flow during her menstrual cycles for the past 3 years. She was symptomatically treated. However, as her symptoms did not subside, she sought further treatment elsewhere, where magnetic resonance imaging (MRI) of the pelvis in August 2017 showed a T1W hypointense, T2W and SPAIR hyper-intense polypoidal mass arising from the cervix measuring 6.2 × 6.9 × 7.2 cm with mild diffusion restriction and the mass was filling the vaginal fornices (Figure 1(A–C)).

A provisional diagnosis of a cervical polyp was made and she underwent a hysteroscopic polypectomy on 8 September 2017. The post-operative histopathology report was in favour of a high grade or poorly differentiated malignancy with the possibility of pleomorphic spindle cell sarcoma/carcinosarcoma (Figure 3 and 4).

**Figure 2(A–C).**
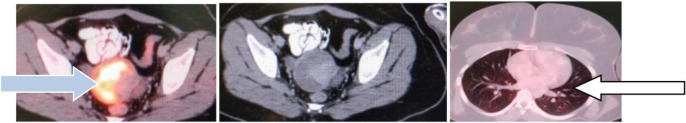
FDG PET CT a 7.0 × 5.5 × 6.3 cm^3^ large exophytic mass arising from uterine cervix and normal lung study done as a part of metastatic work up (physiological uptake in lung).

**Figure 3.**
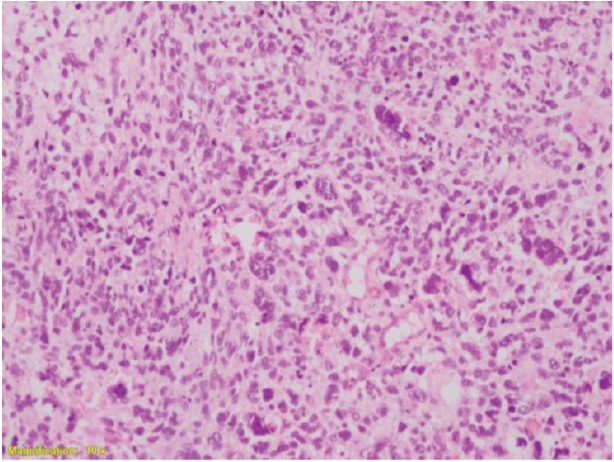
High-grade spindle cell areas with many bizarre giant cells.

**Figure 4.**
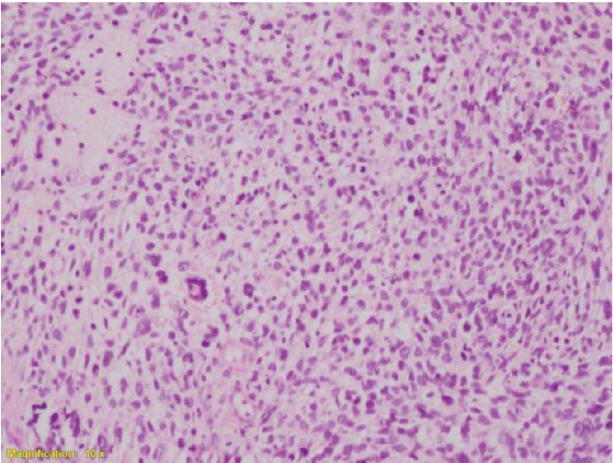
High-grade spindle cell areas with many mitoses.

Fluoro-deoxy-glucose positron emission tomography - computed tomography (FDG-PET CT) done on 9 October 2017 showed an exophytic mass measuring 7.0 × 5.5 × 6.3 cm^3^ arising from the uterine cervix, located predominantly on the proximal vagina with relatively well-defined margins and no evidence of significant regional lymphadenopathy (Figure 2(A–C)).

The hysteroscopic polypectomy slide was reviewed, suggestive of poorly differentiated malignant tumour with high grade sarcomatous content.

**Figure 5 (A–C).**
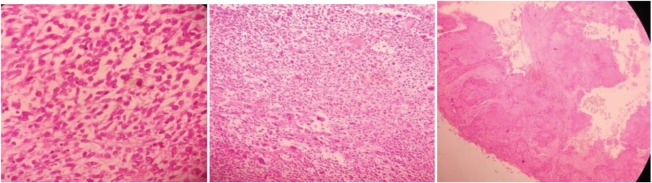
High-grade tumour comprising sheets of plump spindle cells with eosinophilic cytoplasm and enlarged pleomorphic hyperchromatic nuclei.

The patient presented to our hospital in mid-October 2017 with a history of bleeding per vagina and yellowish discharge which on examination revealed a polypoidal fungating necrotic mass measuring 4.0 × 6.0 cm^2^ involving predominantly the anterior lip of the cervix, OS (opening into uterus) was not visualised and bilateral fornices free. She underwent radical hysterectomy type III and bilateral salphingo-oophorectomy with regional pelvic lymph node dissection and omentectomy on 28 October 2017. The post-operative histopathological examination report showed undifferentiated endocervical sarcoma of cervix with all resected lymph nodes, margins and omentum free of tumour (Figure 5(A–C)).

**Figure 6.**
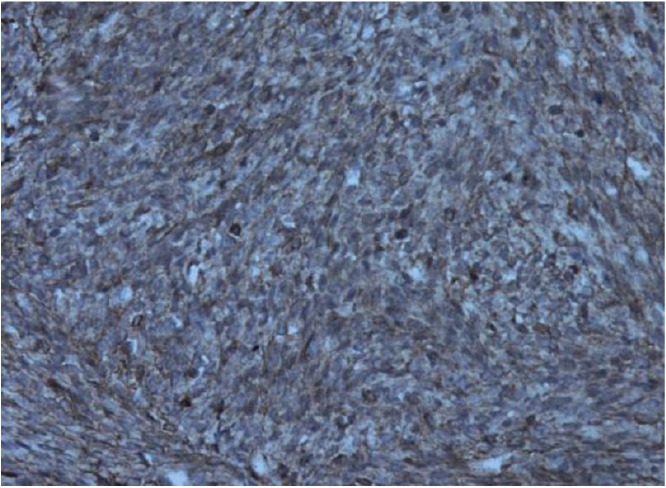
Photomicrograph showing tumour cells showing SMA positivity (200 ×).

**Figure 7.**
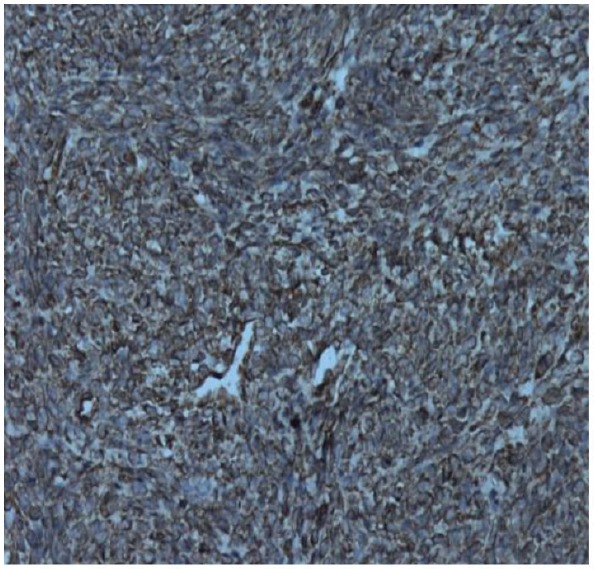
Photomicrograph showing tumour cells showing Vimentin positivity (20 ×).

The slide review of the same procedure by an onco-pathologist revealed pleomorphic leiomyosarcoma of the cervix and the immunohistochemistry was positive for vimentin, smooth muscle actin with Ki 67 of 80% (Figure 6 and 7).

**Figure 8.**
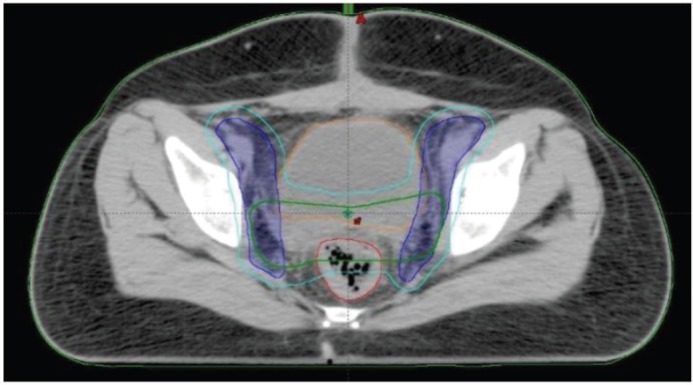
Contoured volumes, cyan-PTV volume, green-CTV tumour volume, blue-CTV nodal volume, red-rectum, orange-urinary bladder.

The patient and her family members were counselled about the condition and treatment options, regarding the role of adjuvant radiotherapy in view of the high degree of proliferative activity and were planned for 50 Gy in 25 fractions followed by adjuvant chemotherapy. The patient has completed adjuvant external beam radiation therapy (EBRT) (Figure 8) and intravaginal brachytherapy with a dose of 6 Gy in three sessions and the patient has received a third cycle of gemcitabine (900 mg/m^2^) and docetaxel (75 mg/m^2^) given every 21st day until the end of March 2018. The patient tolerated the adjuvant treatment without any issue.

## Discussion and review of the literature

The incidence of leiomyosacroma is less than 1% of all uterine malignancies [1]. Leiomyosarcoma represents 0.21% among all invasive tumours of the uterine cervix [2].

Regarding the epidemiology of the tumour, it commonly presents during the 4th–6th decades of life. It is rarely seen in a young age group similar to the patient in our case report [1]. Whitcombe *et al* [3] reported a case in which the patient age was 18 years old and was associated with pregnancy. There are four case reports in which leiomyosarcoma of the uterine cervix was associated with pregnancy [3]. Bansal *et al* [2] analysed the Surveillance Epidemiology and End Results database analysis of tumours of cervix including both carcinoma and sarcoma from 1988 to 2005. A total of 33,074 patients were diagnosed with tumours of carcinoma cervix. Amongst 33,074 patients, 323 patients had sarcoma. Leiomyosarcoma was seen in 67 patients. In this analysis, patients with sarcomas belong to a younger age group, have larger tumours in terms of size and are at a more advanced stage at the time of presentation [2].

Leiomyosarcoma presented with nonpuerperal inversion of the uterus was treated in a 49-year-old nulliparous woman which is an extremely rare event and rare mode of presentation [4]. In contrast to carcinoma cervix, involvement of lymph nodes and parametrium is a very rare event. In view of the rare instances of the involvement of lymph nodes, routine pelvic lymphadenectomy is not recommended, as it does not have any impact on 5-year disease-specific survival. In our patient, pelvic lymphadenectomy was performed in view of the young age of the patient [3].

Ovarian involvement in sarcomas of the cervix, in particular to leiomyosarcoma of the cervix, is a very rare mode of presentation. In view of the rarity of ovarian involvement, the role of oopherectomy in sarcomas of the uterine cervix is a debatable issue. As similar to the other histological variants of sarcoma with some exceptions, the mode of spread to distant sites is through the blood. Lung is the most common site of metastasis [5].

Casanova *et al* [6] reported a case of localised leiomyosarcoma of the cervix in a 63-year-old woman who presented with lung metastasis after 1 year of diagnosis. There are various prognostic factors including tumour stage, grade, mitotic index, tumour size, age and menopausal status. Amongst these factors, stage, grade and mitotic index were more significant prognostic factors [6].

Diagnosis of leiomyosarcoma of the cervix is based on histopathological findings. Microscopic study shows spindle-shaped cells can be seen which may be both hypercellular and hypocellular in nature. The presence of tumour cell necrosis, mitotic index of 10/10 high power field and atypia of cells ranging from moderate to diffuse are histopathological diagnostic criteria [6]. Various morphological varieties of leiomyosarcoma of the cervix myxoid type, epithelioid type, conventional type and certain variants contain abundant xanthomatous cells [7].

There are no well-established risk factors in the aetiology of leiomyosarcoma of the cervix, various risk factors postulated in the aetiology are radiation exposure to the pelvis in childhood; tamoxifen which is an hormonal drug used in breast carcinoma is postulated in the aetiology without any proper explanation for the associated risk; Epstein–Barr virus infection in immunocomprised status is also recognised as an etiological factor without any proper explanation. Patients with abnormal hereditary retinoblastoma gene are also at an increased risk [8].

Signs and symptoms are bleeding per vagina, discharge per vagina, pain and fullness in the pelvic and abdominal region. Most of the tumours are bulky at the time of presentation ranging from 10 to 12 cm in size. Various pressure symptoms such as increased frequency of micturition and sometimes urinary retention can also be seen. The patient is presented with h/o weight loss due to decreased appetite.

Since it is a rare disease, there is no definitive guideline for management of leiomyosarcoma of the cervix. Most recommended treatment is multimodality comprising surgery, total abdominal hysterectomy with bilateral salpingo-oophorectomy in patients having disease confined to the cervix, followed by post-operative radiation therapy to reduce the incidence of local recurrence. In view of the low incidence of lymph node involvement in leiomyosarcoma of the cervix, the role of prophylactic pelvic lymph node dissection along with primary surgery is debatable, unless there is gross pelvic lymph node involvement [1].

The role of chemotherapy has been extrapolated from sarcomas of the uterine corpus; most commonly used drugs including doxorubicin and ifosfamide. This regimen has an overall response rate of 30% in advanced and metastatic forms of disease [1]. Other chemotherapy drugs used are cyclophosphamide, gemcitabine and docetaxel used with response rates of approximately 30% [9].

Radiation therapy is usually given in the adjuvant setting to prevent loco regional recurrence. The pattern of radiation therapy delivered is external beam radiation therapy dose consisting of 50 Gy in 25 fractions over 5 weeks with 2 Gy per fraction followed by brachytherapy consisting of 2–3 sessions of brachytherapy which can also be delivered during EBRT also to reduce the total duration of treatment which has an impact on locoregional control rates [10, 11]. The prognosis of leiomyosarcoma of the cervix is inferior to its other common counterparts such as squamous cell carcinoma and adenocarcinoma [2].

The main treatment modality of this rare histopathological variant involving the cervix is multimodality management consisting of surgery, chemotherapy and radiation therapy. Multimodality management increases the survival rates in such patients [12].

**Table 1.** Literature review of leiomyosarcoma case reports along with treatment modalities and follow-up status.

Author (year of publication)	Treatment protocol	Disease status
Casanova *et al* (2015) [6]	Total abdominal hysterectomy with bilateral salpingo-oophorectomy followed by adjuvant CT.	Patient developed lung metastases and eventually died.
Dhull *et al* (2013) [1]	Simple hysterectomy followed by adjuvant combination chemotherapy (vincristine, adriamycin, cyclophosphamide) followed by radiotherapy.	Asymptomatic without any evidence of tumour recurrence after 6 months of follow-up.
Bhatia *et al* (2015) [12]	Radical hysterectomy, followed by bilateral pelvic lymph node dissection followed by postoperative high-dose vaginal radiotherapy.	Follow-up for a period of 8 months, patient was without evidence of persistent or recurrent disease.
Seema *et al* (2009) [13]	Myomectomy followed by hysterectomy, external beam radiation therapy to the pelvis 50Gy/25F/5 weeks, patient defaulted vault radiation followed by recurrent disease and 3 cycles chemotherapy (palliative) (ifosfamide and doxorubicin).	Diagnosed on 30 October 2004, developed abdominal distention and anorexia expired on 20 August, 2005.
Aminimoghaddam *et al* (2016) [5]	TAH f/b three courses of chemotherapy with docetaxel and gemcitabine f/b metastatic lesion in abdominal wall and cavity f/b two course of chemotherapy with 5-(3,3- dimethyl-1-triazeno)-imidazole-4-carboxamide (DTIC) and adriamycin, f/b debulking surgery and metastatic lesions of sigmoid serous, rectus sheath and bladder serous were resected f/b After ureterolysis, right salpingo-oophorectomy was done because of gross involvement, but the left ovary was spared f/b two course of chemotherapy with 5-(3,3- dimethyl-1-triazeno)-imidazole-4-carboxamide (DTIC) and adriamycin. 6 month later, follow-up CT scan revealed pulmonary nodule and recurrence of disease in bladder and sigmoid. Therefore, the patient received four more courses of chemotherapy with thalidomide, methazolamide and radiotherapy. However, no response was seen, later patient received sunitinib. The disease was stable for 7 months, but imaging revealed pulmonary nodule and the patient received sorafenib.	Last follow-up status not known.
Irvin *et al* (2003) [14]	Modified radical hysterectomy and bilateral salpingo-oophorectomy f/b postoperative high-dose-rate vaginal brachytherapy to the entire vagina.	Without evidence of any disease, patient is on 5th year follow-up at the time of publication of that article.
Whitcombe* et al* (2016) [3]	Primary low transverse cesarean section followed by exploratory laparotomy, total abdominal hysterectomy, and bilateral salpingectomy at 33.5 weeks.	Patient had an uncomplicated postoperative course and has no evidence of recurrent disease by clinical exam and CT scan of the chest, abdomen, and pelvis at 13 months from her diagnosis.
Thambi *et al* (2016) [15]	Total abdominal hysterectomy with debulking of tumour and bilateral salpingo-oophorectomy later advised pelvic radiation and chemotherapy. She disagreed to start the adjuvant therapy.	Patient is now on treatment and she is doing well. No recurrence or metastasis documented (at the time of publication of authors article).
Sahu *et al* (2008) [16]	Two cycles of chemotherapy with cisplatin, doxorubicin and cyclophosphamide f/b total hysterectomy with bilateral salpingo-oophorectomy followed by local radiation to the anterior vaginal wall.	Extensive metastasis within 6 months of surgery.
Toyoshima *et al* (2005) [17]	Total hysterectomy with bilateral salpingo-oophorectomy f/b adjuvant chemotherapy.	Disease-free for over 20 months.
Kasamatsu *et al* (1998) [18]	Hysterectomy with bilateral salpingo-oophorectomy f/b eight courses of combination chemotherapy (VADIC and hydroxyurea, DTIC, etoposide).	Was alive without evidence of recurrence 35 months after the initial therapy.
Khosla *et al* (2012) [11]	Patient took only RT but defaulted the treatment and later presented with disease progression.	Died (10 months).
Khosla *et al* (2012) [11]	Total abdominal hysterectomy with bilateral salpingo-oophorectomy followed by radiation therapy and followed by chemotherapy.	Was alive after 27 months of completion of treatment.
Khosla *et al* (2012) [11]	Total abdominal hysterectomy followed by salpingo-oophorectomy defaulted adjuvant treatment and later came with progressive disease.	Died (11 months after surgery).
Zhiqiang *et al* (2016) [19]	Radical resection of the cervix, bilateral salpingo-oophorectomy and pelvic lymphadenectomy f/b adjuvant chemotherapy and radiotherapy.	Patient suffered from severe menopausal symptoms and received hormone replacement therapy. She eventually committed suicide.
Gotoh *et al* (2001) [20]	Total hysterectomy with bilateral salpingo-oophorectomy.	Patient was alive without any further therapy or complaint 10 months after the initial surgery.

## Conclusion

Leiomyosarcoma of the cervix must be treated with a multimodality management protocol in view of the rarity of the disease, due to the lack of guidelines for its management. Surgery is the main modality of treatment. Radiation therapy in the form of external beam irradiation and intravaginal brachytherapy improves the loco-regional control rate via decreasing the loco-regional recurrence. The role of chemotherapy and drugs to be used are extrapolated from sarcomas of other anatomical areas and sarcoma of the uterine corpus.

## Conflicts of interest

None
